# Safety and Efficacy of Convalescent Plasma Combined with Other Pharmaceutical Agents for Treatment of COVID-19 in Hospitalized Patients: A Systematic Review and Meta-Analysis

**DOI:** 10.3390/diseases12030041

**Published:** 2024-02-21

**Authors:** Massimo Franchini, Daniele Focosi, Mario Cruciani, Michael J. Joyner, Liise-anne Pirofski, Jonathon W. Senefeld, Shmuel Shoham, David J. Sullivan, Arturo Casadevall

**Affiliations:** 1 Department of Transfusion Medicine and Hematology, Carlo Poma Hospital, 46100 Mantua, Italy; crucianimario@virgilio.it; 2 North-Western Tuscany Blood Bank, Pisa University Hospital, 56124 Pisa, Italy; daniele.focosi@gmail.com; 3 Department of Anesthesiology and Perioperative Medicine, Mayo Clinic, Rochester, MN 55905, USA; joyner.michael@mayo.edu; 4 Division of Infectious Diseases, Albert Einstein College of Medicine and Montefiore Medical Center, Bronx, NY 10467, USA; l.pirofski@einsteinmed.edu; 5 Department of Kinesiology and Community Healthy, University of Illinois at Urbana-Champaign, Champaign, IL 61801, USA; senefeld@illinois.edu; 6 Division of Infectious Diseases, Johns Hopkins University School of Medicine, Baltimore, MA 21205, USA; sshoham1@jhmi.edu; 7 Department of Molecular Microbiology and Immunology, Johns Hopkins Bloomberg School of Public Health, Baltimore, MD 21205, USA; dsulliv7@jhmi.edu (D.J.S.); acasade1@jhu.edu (A.C.)

**Keywords:** COVID-19, SARS-CoV-2, convalescent plasma, remdesivir, steroids, combined therapy

## Abstract

Plasma collected from people recovered from COVID-19 (COVID-19 convalescent plasma, CCP) was the first antibody-based therapy employed to fight the pandemic. CCP was, however, often employed in combination with other drugs, such as the antiviral remdesivir and glucocorticoids. The possible effect of such interaction has never been investigated systematically. To assess the safety and efficacy of CCP combined with other agents for treatment of patients hospitalized for COVID-19, a systematic literature search using appropriate Medical Subject Heading (MeSH) terms was performed through PubMed, EMBASE, Cochrane central, medRxiv and bioRxiv. The main outcomes considered were mortality and safety of CCP combined with other treatments versus CCP alone. This review was carried out in accordance with Cochrane methodology including risk of bias assessment and grading of the quality of evidence. Measure of treatment effect was the risk ratio (RR) together with 95% confidence intervals (CIs). A total of 11 studies (8 randomized controlled trials [RCTs] and 3 observational) were included in the systematic review, 4 studies with CCP combined with remdesivir and 6 studies with CCP combined with corticosteroids, all involving hospitalized patients. One RCT reported information on both remdesivir and steroids use with CCP. The use of CCP combined with remdesivir was associated with a significantly reduced risk of death (RR 0.74; 95% CI 0.56–0.97; *p* = 0.03; moderate certainty of evidence), while the use of steroids with CCP did not modify the mortality risk (RR 0.72; 95% CI 0.34–1.51; *p* = 0.38; very low certainty of evidence). Not enough safety data were retrieved form the systematic literature analysis. The current evidence from the literature suggests a potential beneficial effect on mortality of combined CCP plus remdesivir compared to CCP alone in hospitalized COVID-19 patients. No significant clinical interaction was found between CCP and steroids.

## 1. Introduction

During the four-year period December 2019–December 2023, the COVID-19 pandemic caused more than 770 million cases and 7 million deaths worldwide, with an unprecedented global health impact and social crises [1]. Along with oxygen supplementation, the treatment of patients hospitalized for severe COVID-19 initially included the use of repurposed drugs with different mechanisms of action: corticosteroids and tocilizumab for their anti-inflammatory properties, low-molecular-weight heparins for their anti-thrombotic activity, and remdesivir and lopinavir/ritonavir for their antiviral effect [2]. Along with these therapeutic agents, which represented the standard of care during the first months of the pandemic, collection of plasma from individuals who had recovered from SARS-CoV-2 infection (COVID-19 convalescent plasma, CCP) was rapidly deployed around the globe to treat patients with SARS-CoV-2 infection at different stages of disease severity, considering the positive clinical experience in previous viral outbreaks [3]. CCP has been the most intensively studied treatment against COVID-19, and nearly 50 randomized controlled trials (RCTs) have provided evidence to assess its correct place in the anti-COVID-19 therapeutic armamentarium. These studies indicate that CCP has a beneficial clinical effect when administered at high titer (>160) of neutralizing antibodies (nAbs) early (<72 h from symptom onset) in the course of the disease. For immunocompromised patients who are not able to mount a sufficient antibody response after SARS-CoV-2 infection or vaccination in either outpatient or hospital settings, there is evidence that it is also effective at later stages of infection [4,5,6,7]. However, these RCTs rarely used CCP as the sole treatment for COVID-19, but frequently (with rates ranging from 20 to 90 percent) combined CCP with other drugs as part of standard therapy, particularly corticosteroids and remdesivir. In this regard, only a few trials specifically analyzed the possible synergistic or detrimental effects on CCP of such combined agents: a recent systematic review, after a pooled analysis of four studies, found that remdesivir combined with CCP did not have a significantly different effect on mortality compared to remdesivir alone [8]. Therefore, to elucidate this still poorly understood issue, we have conducted a systematic review and meta-analysis analyzing all the published studies on the safety and efficacy of the association between CCP and other anti-SARS-CoV-2 treatments.

## 2. Material and Methods

The aim of this systematic review was to screen all the studies evaluating the clinical effects and adverse reactions to CCP when it was combined with other antiviral agents for the treatment of COVID-19. To perform this type of analysis, patients were classified into two groups: one receiving CCP along with other drugs and another that only received CCP. This systematic review and meta-analysis were developed using the Preferred Reporting Instructions for Systematic Reviews and Meta-analysis (PRISMA) 2020 guidelines [9], and the protocol was pre-registered on PROSPERO (registration number: CRD42023492065).

### 2.1. Literature Search

**Figure 1:**
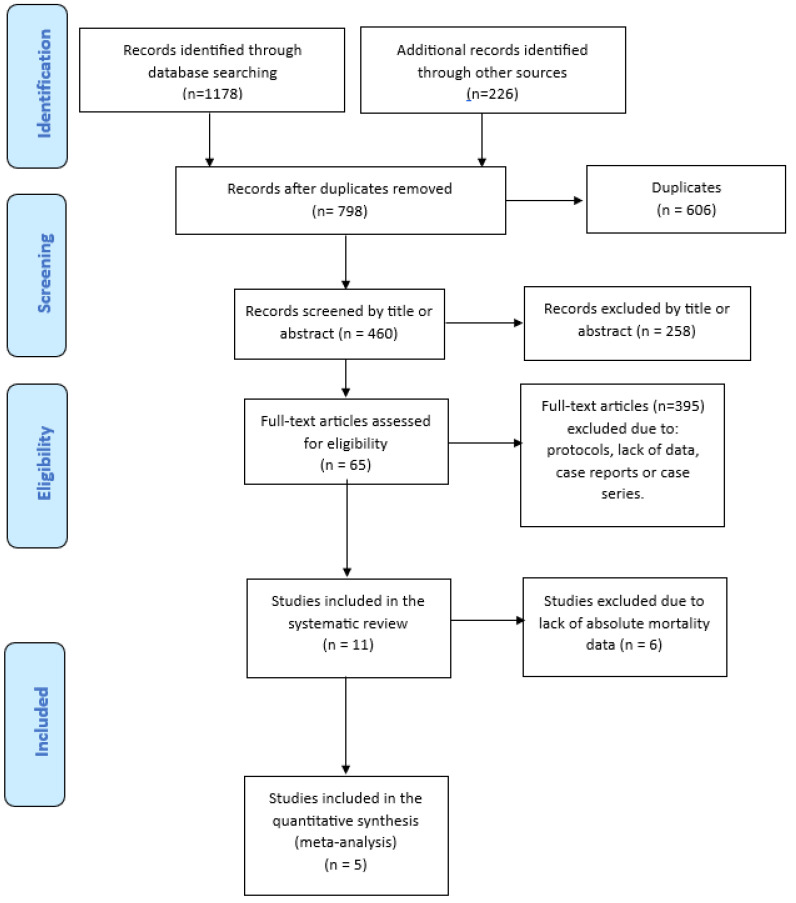
PRISMA flow diagram of literature studies selection.

A literature search of PubMed (through Medline), EMBASE, Cochrane Central, medRxiv and bioRxiv databases was carried out between January 2020 and December 2023, using the English language as a restriction. The Medical Subject Heading (MeSH) and search query used were: “(“COVID-19” OR “SARS-CoV-2” OR “coronavirus disease 2019”) AND (“COVID-19 convalescent plasma” OR “hyperimmune plasma” OR “combined therapy” OR “combination” OR “association” OR “lopinavir/ritonavir” OR “remdesivir” OR “steroids” OR “glucocorticoids” OR “hydroxychloroquine” OR “tocilizumab”) AND (“efficacy” OR “mortality” OR “death” OR “safety” OR “adverse reactions” OR “infusion related” OR “allergic reactions”). We also screened the reference list of all retrieved studies and of review articles for additional studies not captured in our initial literature search. Finally, a PRISMA flowchart of the literature reviewing process was produced (Figure 1).

### 2.2. Types of Studies, Participants, Interventions and Outcome Measures Included

Only RCTs and observational (prospective and retrospective) studies assessing the safety and efficacy of CCP associated with other treatments were included in this systematic review. We excluded case reports, case series, review articles, meta-analyses and original research articles reporting only aggregate data. We included individuals with a confirmed diagnosis of COVID-19, with no gender, age or ethnicity restrictions. We included studies that enrolled hospitalized or ambulatory patients with any COVID-19 disease severity. We defined CCP in combination with other drugs as the intervention and CCP alone as the control. Studies evaluating CCP combined with other passive immunotherapies (i.e., anti-Spike monoclonal antibodies and hyperimmune immunoglobulins against COVID-19) were not included in this systematic review. Each drug associated with CCP was analyzed independently from the others. Mortality was set as the primary outcome measure for this analysis, and only studies providing information on survival in both intervention and control groups were included in this systematic review. Of them, those studies reporting absolute mortality data were also included in the quantitative meta-analysis. Adverse events were graded according to the Common Terminology Criteria for Adverse Events, version 6.0 (available at Common Terminology Criteria for Adverse Events (CTCAE)|Protocol Development|CTEP (cancer.gov) last access: 10 December 2023).

The following parameters were extracted from each study: study design and, for each arm, patients enrolled, outcome and safety data. Articles underwent a blind evaluation for inclusion by two assessors (M.F. and D.F.), and disagreements were resolved by a third senior assessor (M.C.).

### 2.3. Quality Assessment

We have considered both RCTs and controlled non-RCTs. Within-trial risk of bias is assessed using the Cochrane ROB tool for RCTs and the ROBINS-I tool for non-RCTs. The Cochrane “risk of bias” tool for RCTs addresses six specific domains: sequence generation, allocation concealment, blinding, incomplete data, selective outcome reporting and other issues relating to bias [10]. The methodological quality of observational studies was assessed with the ROBINS-1 tool [11]. This tool includes 7 specific bias domains: (1) confounding; (2) selection of participants; (3) classification of intervention; (4) deviation from interventions (biases that arise when there are systematic differences between the care provided to experimental intervention and comparator groups, beyond the assigned interventions); (5) missing outcome; (6) measurement of outcomes (blinding of outcome assessors aims to prevent systematic differences in measurements between intervention groups, but it is less common in non-RCTs than in RCTs; (7) selection of reported result overall. 

We used the principles of the GRADE (The Grading of Recommendations Assessment, Development and Evaluation) system to assess the quality of the body of evidence associated with outcomes and constructed a “Summary of findings table” presenting key information concerning the certainty of the evidence, the magnitude of the effects of the interventions examined and the sum of available data for the main outcomes [12,13]. The certainty of a body of evidence involves consideration of within-trial risk of bias (methodological quality), directness of evidence, heterogeneity and precision of effect estimates. Due to the low number of available studies, publication bias was not assessed.

### 2.4. Statistical Analysis

Descriptive statistics included reporting of continuous variables as mean (SD) or median (range) as appropriate according to distribution, while categorical variables were reported as numbers and percentages. For each trial, we compared the observed number of deaths at 30 days (or the closest available time point) of patients allocated to the CCP combined therapy group or to the CCP-alone group. The treatment effect was measured as risk ratio (RR) and 95% confidence intervals (CIs). The study weight was calculated using the Mantel–Haenszel method. Clinical heterogeneity was quantified using the inconsistency index (*I*^2^), which explores the percentage of total variation across studies that is due to heterogeneity rather than to chance, and *p*-values from the chi-square test for homogeneity. If significant heterogeneity was detected, a random effect method of study weight calculation was performed.

## 3. Results

### 3.1. Study Flow Diagram

A total of 1404 studies were initially identified after electronic database and manual search. After the removal of 606 duplicates, we screened the titles and abstracts of 460 studies. The screening of the full text of such articles led to the exclusion of a further 395 studies, resulting in 65 articles assessed for eligibility. Finally, 11 studies [14,15,16,17,18,19,20,21,22,23,24], including 8 RCTs and 3 observational studies, were included in the systematic review and 5 of them (3 observational and 2 RCTs) in the meta-analysis. The process of study selection is represented in the PRISMA flow diagram (Figure 1). 

### 3.2. Study Characteristics

All studies involved hospitalized COVID-19 patients. Among the various repurposed drugs identified in the systematic search, only remdesivir and steroids were combined with CCP at baseline, and outcomes were reported separately. One RCT (CONTAIN COVID-19) [18] analyzed CCP in association with steroids or remdesivir and thus was analyzed in both CCP + remdesivir and CCP + steroids groups. The main characteristics of studies included in the systematic review are summarized in Table 1.

### 3.3. Mortality Outcome

Among the 11 studies included in the systematic review, 5 studies (3 observational and 2 RCTs) evaluated CCP alone or combined with remdesivir (CCP + remdesivir group), and 6 RCTs (plus the CONTAIN COVID-19 RCT) assessed CCP alone or combined with steroids. 

Considering the remdesivir group, two non-RCTs [15,16] reported a reduced mortality rate in favor of the combined treatment arm, and two other studies (an observational trial [14] and an RCT [18]) found a slight, non-significant, decreased mortality rate in the CCP-alone arm. In the TSUNAMI RCT [17], the mortality rate was included in the composite primary outcome, which was found similar between CCP-alone or combined groups.

Regarding the CCP + steroid group, five RCTs [18,21,22,23,24] observed a favorable outcome (i.e., mortality or clinical status) in patients receiving only CCP versus combined (CCP + steroids) therapy, while the others [19,20] found no interaction. Unfortunately, it was not possible to retrieve patients’ demographic and clinical information as these types of data were not available from subgroup analyses of single studies.

### 3.4. Safety

Three studies evaluating CCP with or without remdesivir performed a safety analysis. While Moniuszko [14] reported no adverse effects, Koirala [15] and Arquiette [16] reported an incidence of treatment-related adverse effects of 5% and 10%, respectively. No safety data are available regarding studies on CCP with or without steroids.

### 3.5. Meta-Analysis

**Figure 2:**
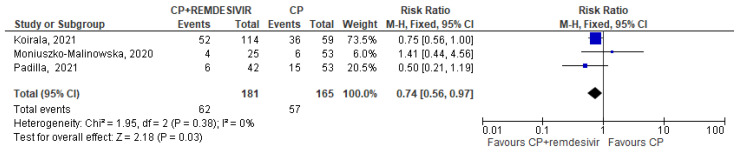
Forest plot of comparison: convalescent plasma plus remdesivir versus convalescent plasma alone, outcome: mortality [14,15,16].

**Figure 3:**
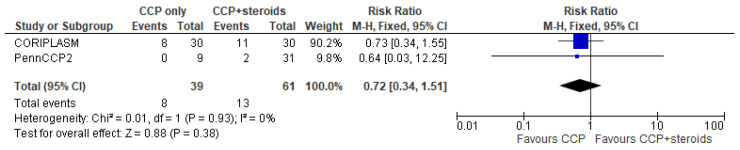
Forest plot of comparison: convalescent plasma plus steroids versus convalescent plasma alone, outcome: mortality [22,24].

Only 5 trials (three in the CCP + remdesivir group and two in the CCP + steroids group) analyzed separately the mortality rate in both treatment and control arms and permitted to perform a pooled quantitative analysis [14,15,16,22,24]. Regarding the three studies [14,15,16] evaluating CCP alone or combined with remdesivir, the forest plot of comparison showed that the association CCP + remdesivir significantly reduced the mortality risk compared to CCP alone (RR 0.74; 95% CI 0.56–0.97; *I^2^* 0%, *p* = 0.03) (Figure 2). Regarding the two studies [22,24] included in the meta-analysis on CCP combined with steroids, no significant differences were found between CCP + steroids recipients and CCP-alone recipients (RR 0.72; 95% CI 0.34–1.51; *I^2^* 0%, *p* = 0.38) (Figure 3).

### 3.6. Certainty of Evidence

The final certainty of the available evidence with GRADE assessment (Summary of findings table [SOT], Table 2) showed a moderate certainty level within CCP + remdesivir trials and very low certainty with CCP + steroids trials. The main reason for downgrading studies with CCP + remdesivir was ROB in non-RCTs (mostly confounding bias). The very low certainty with CCP + steroids trials is related to serious imprecision (low number of participants and wide intervals of confidence around the effect) and indirectness (the evidence is restricted to indirect comparisons between CCP and CCP + steroids recipients and is not related to the relevance of the included studies to the research question). CCP + steroids trials were unblinded but were not downgraded for ROB because masking probably has limited importance for the outcome’s mortality compared to other subjective outcomes. Publication bias was not evaluated due to the low number of relevant trials.

## 4. Discussion

CCP was the first antibody-based therapy used during the COVID-19 pandemic outbreak. However, only rarely was it used as a single therapeutic agent. More frequently, including in RCTs, it was used in combination with other small-molecule therapeutics. However, only a few studies assessed the possible effect of this interaction and among the various drugs combined with CCP as part of the standard antiviral therapy, only remdesivir and steroids were analyzed in detail by a few studies whose results are reported in this systematic review, the first performed so far on this issue. 

For both small-molecule antivirals and corticosteroids, there is both theoretical and experimental evidence that these could influence antibody action. Such combinations could be additive or synergistic, since the mechanisms of action for small-molecule antiviral (inhibition of replication) and antibody (virion neutralization) are independent and should not interfere with each other. CCP could also add an anti-inflammatory effect via Fc functional activity [25]. In this regard, additive or synergistic effects were observed for combination therapy with acyclovir and a specific antibody to the Herpes simplex virus in experimental animals [26,27]. For remdesivir, there is some evidence that combination with a specific antibody was more effective against Sudan viral disease in non-human primates [28] and SARS-CoV-2 in patients with COVID-19 [29,30]. In contrast, corticosteroids are known to interfere with such Fc-mediated functions and antibody-dependent cellular cytotoxicity (ADCC) [31] and phagocytosis [32], which have been shown to be important for CCP efficacy [33]. In this regard, concern has been raised that the negative results with some RCTs of CCP could have been a result of concomitant dexamethasone usage [34]. 

Although the certainty of evidence was moderate (due to the risk of bias linked to non-RCTs), the results of this meta-analysis suggest a potential clinical benefit of CCP combined with remdesivir in patients hospitalized for COVID-19. This synergic effect is particularly intriguing and interesting in light of the current pandemic situation, where omicron variants are not dangerous for the general COVID-19-vaccinated population but are potentially life-threatening for immunocompromised people. Besides oral small-molecule antivirals, such patients often need antibody-based therapies (CCP is the only effective antibody-based therapy currently available) to clear the virus because they are rarely able to mount an efficient antibody response following SARS-CoV2 vaccination or infection. The results of this meta-analysis are consistent with those from a number of case series and case reports that reported the successful combined use of CCP and remdesivir [35,36,37,38,39,40,41,42,43,44], particularly in B-cell-depleted onco-hematologic patients who could represent a peculiar subgroup of COVID-19 patients who could take particular advantage from the association of antiviral and antibody-based therapies. 

The association of CCP with steroids against COVID-19 was also investigated. Unfortunately, all but two of the seven RCTs selected in the systematic review included mortality as part of a composite primary outcome evaluating also other parameters, and thus, the performed aggregated analyses did not allow us to calculate mortality (our prespecified outcome) separately in patients receiving CCP with and without steroids. Thus, the pooled quantitative analysis included only two small RCTs with a very low grade of certainty (for serious imprecision and indirectness) and no statistically significant difference between treatment and control arms. Thus, while the association of remdesivir to CCP seems to enhance its antiviral effect (a finding which is not surprising considering the different antiviral mechanisms of action of small molecules and antibodies), no conclusions can be drawn regarding the possible effects of the interaction between CCP and steroids. As an exclusively speculative consideration, the observation from Table 1 that five out of seven studies found the worst outcome in CCP + steroids compared to CCP alone might suggest a potential detrimental consequence of such a combination that warrants further investigation.

Several study limitations, in addition to the risk of bias linked to the inclusion of non-RCT data, exist, which can be grouped as either limitations in studies included in the systematic review or limitations of the methodology of this systematic review. Among the first, there was great heterogeneity in the determination of neutralizing antibody titers across the included studies. Among the latter, the number of included studies was overall limited, and we could not take into account detailed demographics of individual studies (e.g., age or risk factors for COVID-19 progression).

## 5. Conclusions

While no definitive conclusions can be drawn regarding the safety of the combination of CCP with remdesivir or steroids due to the paucity or lack of data from trials analyzed, the suggestion of benefit for the combination of remdesivir and CCP is consistent with literature reports that combinations of antivirals and antibodies can be more effective than either agent. Further studies, randomizing COVID-19 patients to receive CCP combined or not with other drugs, are therefore needed to better assess the clinical effect and the safety of these combinations.

## Figures and Tables

**Table 1:** Main characteristics of the studies included in the systematic review.

Author, State, Year [Ref.]	Study Design	Patients Enrolled (*n*)	Outcome (Death)	Safety
**CCP plus remdesivir**				
Moniuszko-Malinowska, Poland, 2020 [14]	Prospective	78 patients hospitalized for COVID-19 - CCP + remdesivir: 25/78 (32.1%) - CCP: 53/78 (67.9%)	30-day mortality: - CCP + remdesivir: 4/25 (16.0%) - CCP: 6/53 (11.3%) No difference was observed in the duration of hospitalization, the necessity of mechanical ventilation, or the duration of oxygen therapy between the two groups.	No side effects were recorded.
Koirala, Nepal, 2021 [15]	Prospective	1083 patients hospitalized for moderate, severe, or life-threatening COVID-19 - CCP + remdesivir: 114/173 (65.9%) - CCP: 59/173 (34.1%)	Mortality: - CCP + remdesivir: 52/114 (45.6%) - CCP: 36/59 (61.0%)	Adverse events to remdesivir and CCP were reported in 4.5% (increased liver enzymes and serum creatinine levels) and 4.6% (fever, rash) of patients, respectively.
Arquiette, USA, 2021 [16]	Retrospective	213 patients hospitalized for COVID-19 - CCP + remdesivir: 42/95 (44.2%) - CCP: 53/95 (55.8%)	Mortality: - CCP + remdesivir: 6/42 (14.3%) - CCP: 15/53 (28.3%) Patients with CCP monotherapy had decreased ventilation days and duration of hospitalization compared to those treated with combination therapy.	Six adverse (4 increased liver enzymes and 2 serum creatinine levels) reactions were recorded with remdesivir, 2 adverse reactions after CCP infusion.
TSUNAMI, Italy, 2021 [17]	RCT	487 patients hospitalized with COVID-19 pneumonia. - CCP + remdesivir: 157/232 (67.7%) - CCP: 74/232 (31.9%)	Composite of worsening respiratory failure (PaO_2_/FiO_2_ ratio < 150 mmHg) or death within 30 days from randomization (primary endpoint): - CCP + remdesivir: 42/157 (26.8%) - CCP: 17/74 (23.0%)	NA
CONTAIN COVID-19, USA, 2021 [18]	RCT	941 patients hospitalized with COVID-19 - CCP + remdesivir: 273/468 (58.3%) - CCP: 195/468 (41.7%)	The OR for mortality at day 28 for CCP alone was lower than that for CCP + remdesivir (0.77; 95% CI 0.43–1.36 versus 1.0; 95% CI 0.52–1.96).	NA
**CCP plus corticosteroids**				
CONTAIN COVID-19, USA, 2021 [18]	RCT	941 patients hospitalized with COVID-19 - CCP + steroids: 407/468 (87.0%) - CCP: 61/468 (13.0%)	The OR for mortality at day 28 for CCP alone was lower than that for CCP + steroids (0.77; 95% CI 0.43–1.36 versus 0.86; 95% CI 0.49–1.52).	NA
PlasmAr, Argentina, 2021 [19]	RCT	333 patients hospitalized with COVID-19 - CCP + steroids: 209/228 (91.7%) - CCP: 19/228 (8.3%)	No interaction between CCP and steroids was found.	NA
CONCOR-1, Canada, 2021 [20]	RCT	940 patients hospitalized with COVID-19 - CCP + steroids: 496/614 (80.8%) - CCP alone: 118/614 (19.2%)	Primary outcome (intubation or death by 30 days): - CCP + steroids: 159/496 (32.1%) - CCP alone: 40/118 (33.9%)	NA
Conplas-19, Spain, 2021 [21]	RCT	350 patients hospitalized with COVID-19 - CCP + steroids: 127/179 (70.9%) - CCP alone: 52/179 (29.1%)	Outcome (primary and secondary) at 14 days: - CCP + steroids: 18/127 (14.2%) - CCP alone: 3/52 (5.8%) Outcome (primary and secondary) at 28 days: - CCP + steroids: 12/127 (9.5%) - CCP alone: 3/52 (5.8%)	NA
PennCCP2, USA, 2021 [22]	RCT	79 patients hospitalized with COVID-19 - CCP + steroids: 31/40 (77.5%) - CCP alone: 9/40: 22.5%)	Mortality at 28 days: - CCP + steroids: 2/31 (6.5%) - CCP alone: 0/9	NA
RECOVERY, UK, 2021 [23]	RCT	11,558 patients hospitalized with COVID-19 - CCP + steroids: 5370/5795 (92.7%) - CCP alone: 391/5795 (6.7%)	Outcome (28-day invasive mechanical ventilation or death) - CCP + steroids: 1491/5100 (29.2%) - CCP alone: 66/360 (18.3%)	NA
CORIPLASM, France, 2023 [24]	RCT	120 patients hospitalized with COVID-19 - CCP + steroids: 30/60 (50.0%) - CCP alone: 30/60 (50.0%)	Outcome (14-day mortality) - CCP + steroids: 11/30 (36.7%) - CCP alone: 8/30 (26.7%)	NA

Abbreviations: RCT, randomized controlled trial; CCP, COVID-19 convalescent plasma; NA, not available; OR, odds ratio.

**Table 2:** Summary of findings table.

Settings: Hospitalized Patients with COVID-19 Intervention: Convalescent Plasma (CCP) Comparison: CCP + Remdesivir, CCP + Steroids						
**Outcome:** **Mortality**	Illustrative comparative risks * (95% CI)		Relative effect (95% CI)	No. of Participants(studies)	Quality of the evidence (GRADE)	Comments
	Assumed risk	Corresponding risk				
	Controls (CCP + Other)	Intervention (CCP)				
**CCP+ Remdesivir**	342 per 1000	253 per 1000 (from 191 to 331)	RR 0.74 (95% CIs, 0.56 to 0.97)	346 patients from 3 non-RCTs	Moderate. Downgraded twice for nonserious ROB	CCP in combination with remdesivir had a lower mortality rate compared to CCP alone.
**CCP+ steroids**	639 per 1000	460 per 1000 (from 217 to 964)	RR 0.72 (0.34/1.51)	100 patients from 2 RCTs	Very low. Downgraded for serious imprecision and indirectness	Rates of mortality were not significantly different in CCP recipients compared to CCP + steroids recipients.
* The basis for the assumed risk is the mean control group risk across studies. The corresponding risk (and its 95% confidence interval) is based on the assumed risk in the comparison group and the relative effect of the intervention (and its 95% CI). CI: Confidence interval; RR: risk ratio						
GRADE Working Group grades of evidence High quality: Further research is very unlikely to change our confidence in the estimate of effect. Moderate quality: Further research is likely to have an important impact on our confidence in the estimate of effect and may change the estimate. Low quality: Further research is very likely to have an important impact on our confidence in the estimate of effect and is likely to change the estimate. Very low quality: We are very uncertain about the estimate.						

## Data Availability

This manuscript generated no novel dataset.

## References

[1] WHO Coronavirus Disease (COVID-19) Pandemic. https://www.who.int/emergencies/diseases/novel-coronavirus-2019.

[2] Yuan Y., Jiao B., Qu L., Yang D., Liu R. (2023). The development of COVID-19 treatment. Front. Immunol..

[3] Casadevall A., Pirofski L.A. (2020). The convalescent sera option for containing COVID-19. J. Clin. Investig..

[4] Senefeld J.W., Franchini M., Mengoli C., Cruciani M., Zani M., Gorman E.K., Focosi D., Casadevall A., Joyner M.J. (2023). COVID-19 Convalescent Plasma for the Treatment of Immunocompromised Patients: A Systematic Review and Meta-analysis. JAMA Netw. Open.

[5] Senefeld J.W., Gorman E.K., Johnson P.W., Moir M.E., Klassen S.A., Carter R.E., Paneth N.S., Sullivan D.J., Morkeberg O.H., Wright R.S. (2023). Rates Among Hospitalized Patients with COVID-19 Treated with Convalescent Plasma: A Systematic Review and Meta-Analysis. Mayo Clin. Proc. Innov. Qual. Outcomes.

[6] Bloch E.M., Focosi D., Shoham S., Senefeld J., Tobian A.A.R., Baden L.R., Tiberghien P., Sullivan D.J., Cohn C., Dioverti V. (2023). Guidance on the Use of Convalescent Plasma to Treat Immunocompromised Patients with Coronavirus Disease 2019. Clin. Infect. Dis..

[7] Sullivan D.J., Focosi D., Hanley D.F., Cruciani M., Franchini M., Ou J., Casadevall A., Paneth N. (2023). Outpatient regimens to reduce COVID-19 hospitalisations: A systematic review and meta-analysis of randomized controlled trials. medRxiv.

[8] Chen C., Fang J., Chen S., Rajaofera M.J.N., Li X., Wang B., Xia Q. (2023). The efficacy and safety of remdesivir alone and in combination with other drugs for the treatment of COVID-19: A systematic review and meta-analysis. BMC Infect. Dis..

[9] Page M.J., McKenzie J.E., Bossuyt P.M., Boutron I., Hoffmann T.C., Mulrow C.D., Shamseer L., Tetzlaff J.M., Akl E.A., Brennan S.E. (2021). The PRISMA 2020 statement: An updated guideline for reporting systematic reviews. BMJ.

[10] Higgins J.P., Green S. (2011). Cochrane Handbook for Systematic Reviews of Interventions.

[11] Sterne J.A., Hernán M.A., Reeves B.C., Savović J., Berkman N.D., Viswanathan M., Henry D., Altman D.G., Ansari M.T., Boutron I. (2016). ROBINS-I: A tool for assessing risk of bias in non-randomised studies of interventions. BMJ.

[12] Schünemann H.J., Oxman A.D., Higgins J.P., Vist G.E., Glasziou P., Guyatt G.H., Higgins J.P., Green S. (2011). Chapter 11: Presenting results and ’Summary of findings’ tables. Cochrane Handbook for Systematic Reviews of Interventions.

[13] Guyatt G.H., Oxman A.D., Kunz R., Vist G.E., Falck-Ytter Y., Schünemann H.J. (2008). What is ‘quality of evidence’ and why is it important to clinicians?. BMJ.

[14] Moniuszko-Malinowska A., Czupryna P., Zarębska-Michaluk D., Tomasiewicz K., Pancewicz S., Rorat M., Dworzańska A., Sikorska K., Bolewska B., Lorenc B. (2020). Convalescent Plasma Transfusion for the Treatment of COVID-19-Experience from Poland: A Multicenter Study. J. Clin. Med..

[15] Koirala J., Gyanwali P., Gerzoff R.B., Bhattarai S., Nepal B., Manandhar R., Jha R., Sharma S., Sharma Y.R., Bastola A. (2021). Experience of Treating COVID-19 with Remdesivir and Convalescent Plasma in a Resource-Limited Setting: A Prospective, Observational Study. Open Forum Infect. Dis..

[16] Arquiette J., Padilla R., Mai Y., Singh G., Galang K., Liang E. (2021). Clinical Outcomes of COVID-19 Patients Treated with Convalescent Plasma or Remdesivir Alone and in Combination at a Community Hospital in California’s Central Valley. J. Pharm. Pharm. Sci..

[17] Menichetti F., Popoli P., Puopolo M., Spila Alegiani S., Tiseo G., Bartoloni A., De Socio G.V., Luchi S., Blanc P., Puoti M. (2021). Effect of High-Titer Convalescent Plasma on Progression to Severe Respiratory Failure or Death in Hospitalized Patients with COVID-19 Pneumonia: A Randomized Clinical Trial. JAMA Netw. Open.

[18] Ortigoza M.B., Yoon H., Goldfeld K.S., Troxel A.B., Daily J.P., Wu Y., Li Y., Wu D., Cobb G.F., Baptiste G. (2022). Efficacy and Safety of COVID-19 Convalescent Plasma in Hospitalized Patients: A Randomized Clinical Trial. JAMA Intern. Med..

[19] Libster R., Pérez Marc G., Wappner D., Coviello S., Bianchi A., Braem V., Esteban I., Caballero M.T., Wood C., Berrueta M. (2021). Early High-Titer Plasma Therapy to Prevent Severe COVID-19 in Older Adults. N. Engl. J. Med..

[20] Bégin P., Callum J., Jamula E., Cook R., Heddle N.M., Tinmouth A., Zeller M.P., Beaudoin-Bussières G., Amorim L., Bazin R. (2021). Convalescent plasma for hospitalized patients with COVID-19: An open-label, randomized controlled trial. Nat. Med..

[21] Avendaño-Solá C., Ramos-Martínez A., Muñez-Rubio E., Ruiz-Antorán B., Malo de Molina R., Torres F., Fernández-Cruz A., Calderón-Parra J., Payares-Herrera C., Díaz de Santiago A. (2021). A multicenter randomized open-label clinical trial for convalescent plasma in patients hospitalized with COVID-19 pneumonia. J. Clin. Investig..

[22] Bar K.J., Shaw P.A., Choi G.H., Aqui N., Fesnak A., Yang J.B., Soto-Calderon H., Grajales L., Starr J., Andronov M. (2021). A randomized controlled study of convalescent plasma for individuals hospitalized with COVID-19 pneumonia. J. Clin. Investig..

[23] RECOVERY Collaborative Group (2021). Convalescent plasma in patients admitted to hospital with COVID-19 (RECOVERY): A randomised controlled, open-label, platform trial. Lancet.

[24] Lacombe K., Hueso T., Porcher R., Mekinian A., Chiarabini T., Georgin-Lavialle S., Ader F., Saison J., Martin-Blondel G., De Castro N. (2023). Use of covid-19 convalescent plasma to treat patients admitted to hospital for covid-19 with or without underlying immunodeficiency: Open label, randomised clinical trial. BMJ Med..

[25] Herman J.D., Wang C., Loos C., Yoon H., Rivera J., Eugenia Dieterle M., Haslwanter D., Jangra R.K., Bortz R.H., Bar K.J. (2021). Functional convalescent plasma antibodies and pre-infusion titers shape the early severe COVID-19 immune response. Nat. Commun..

[26] Hilfenhaus J., De Clercq E., Köhler R., Geursen R., Seiler F. (1987). Combined antiviral effects of acyclovir or bromovinyldeoxyuridine and human immunoglobulin in herpes simplex virus-infected mice. Antivir. Res..

[27] Bravo F.J., Bourne N., Harrison C.J., Mani C., Stanberry L.R., Myers M.G., Bernstein D.I. (1996). Effect of antibody alone and combined with acyclovir on neonatal herpes simplex virus infection in guinea pigs. J. Infect. Dis..

[28] Cross R.W., Bornholdt Z.A., Prasad A.N., Woolsey C., Borisevich V., Agans K.N., Deer D.J., Abelson D.M., Kim D.H., Shestowsky W.S. (2022). Combination therapy with remdesivir and monoclonal antibodies protects nonhuman primates against advanced Sudan virus disease. JCI Insight.

[29] Scotto R., Buonomo A.R., Iuliano A., Foggia M., Sardanelli A., Villari R., Pinchera B., Gentile I., Federico II COVID-Team (2023). Remdesivir Alone or in Combination with Monoclonal Antibodies as an Early Treatment to Prevent Severe COVID-19 in Patients with Mild/Moderate Disease at High Risk of Progression: A Single Centre, Real-Life Study. Vaccines.

[30] Hirai J., Mori N., Sakanashi D., Ohashi W., Shibata Y., Asai N., Kato H., Hagihara M., Mikamo H. (2023). Real-World Experience of the Comparative Effectiveness and Safety of Combination Therapy with Remdesivir and Monoclonal Antibodies versus Remdesivir Alone for Patients with Mild-to-Moderate COVID-19 and Immunosuppression: A Retrospective Single-Center Study in Aichi, Japan. Viruses.

[31] Nair M.P., Schwartz S.A. (1984). Immunomodulatory effects of corticosteroids on natural killer and antibody-dependent cellular cytotoxic activities of human lymphocytes. J. Immunol..

[32] Jones C.J., Morris K.J., Jayson M.I. (1983). Prednisolone inhibits phagocytosis by polymorphonuclear leucocytes via steroid receptor mediated events. Ann. Rheum. Dis..

[33] Ullah I., Beaudoin-Bussières G., Symmes K., Cloutier M., Ducas E., Tauzin A., Laumaea A., Grunst M.W., Dionne K., Richard J. (2023). The Fc-effector function of COVID-19 convalescent plasma contributes to SARS-CoV-2 treatment efficacy in mice. Cell Rep. Med..

[34] Casadevall A., Sullivan D.J. (2024). Late administration and corticosteroid usage explain inefficacy in COVID-19 convalescent plasma trial. J. Infect. Dis..

[35] Anderson J., Schauer J., Bryant S., Graves C.R. (2020). The use of convalescent plasma therapy and remdesivir in the successful management of a critically ill obstetric patient with novel coronavirus 2019 infection: A case report. Case Rep. Women Health.

[36] Magyari F., Pinczés L.I., Páyer E., Farkas K., Ujfalusi S., Diószegi Á., Sik M., Simon Z., Nagy G., Hevessy Z. (2022). Early administration of remdesivir plus convalescent plasma therapy is effective to treat COVID-19 pneumonia in B-cell depleted patients with hematological malignancies. Ann. Hematol..

[37] Malsy J., Veletzky L., Heide J., Hennigs A., Gil-Ibanez I., Stein A., Lütgehetmann M., Rosien U., Jasper D., Peine S. (2021). Sustained Response After Remdesivir and Convalescent Plasma Therapy in a B-Cell-Depleted Patient with Protracted Coronavirus Disease 2019 (COVID-19). Clin. Infect. Dis..

[38] Dell’Isola G.B., Felicioni M., Ferraro L., Capolsini I., Cerri C., Gurdo G., Mastrodicasa E., Massei M.S., Perruccio K., Brogna M. (2021). Case Report: Remdesivir and Convalescent Plasma in a Newly Acute B Lymphoblastic Leukemia Diagnosis with Concomitant Sars-CoV-2 Infection. Front. Pediatr..

[39] Weinbergerova B., Mayer J., Kabut T., Hrabovsky S., Prochazkova J., Kral Z., Herout V., Pacasova R., Zdrazilova-Dubska L., Husa P. (2021). Successful early treatment combining remdesivir with high-titer convalescent plasma among COVID-19-infected hematological patients. Hematol. Oncol..

[40] Jamir I., Lohia P., Pande R.K., Setia R., Singhal A.K., Chaudhary A. (2020). Convalescent plasma therapy and remdesivir duo successfully salvaged an early liver transplant recipient with severe COVID-19 pneumonia. Ann. Hepatobiliary Pancreat. Surg..

[41] Schenker C., Hirzel C., Walti L.N., Zeerleder S.S., Andres M., Ramette A., Barbani M.T., Suter-Riniker F., Holbro A., Tritschler T. (2022). Convalescent plasma and remdesivir for protracted COVID-19 in a patient with chronic lymphocytic leukaemia: A case report of late relapse after rapid initial response. Br. J. Haematol..

[42] Raho G., Cordeddu W., Firinu D., Del Giacco S.W., Angioni G. (2023). Successful combination of remdesivir and convalescent plasma to treat a patient with rituximab-related B-cell deficiency and prolonged COVID-19: A case report. Anti-Infect. Agents.

[43] Iaboni A., Wong N., Betschel S.D. (2021). A Patient with X-Linked Agammaglobulinemia and COVID-19 Infection Treated with Remdesivir and Convalescent Plasma. J. Clin. Immunol..

[44] Furlan A., Forner G., Cipriani L., Vian E., Rigoli R., Gherlinzoni F., Scotton P. (2021). Dramatic Response to Convalescent Hyperimmune Plasma in Association with an Extended Course of Remdesivir in 4 B Cell-Depleted Non-Hodgkin Lymphoma Patients with SARS-Cov-2 Pneumonia After Rituximab Therapy. Clin. Lymphoma Myeloma Leuk..

